# Splicing factor PTBP1 promotes hepatocarcinogenesis via oncogenic splice-switching of MAPT

**DOI:** 10.32604/or.2025.060958

**Published:** 2025-04-18

**Authors:** WENYING ZHENG, YANYAN SHANG, KAI DU, AILING LUO, LIJUN PEI, MEIQI LI, GUOPING ZHANG, MIN DENG

**Affiliations:** 1 Guangzhou Institute of Cancer Research, The Affiliated Cancer Hospital, Guangzhou Medical University, Guangzhou, 510095, China; 2 Medical Oncology, Yuebei People Hospital, Shaoguan, 512026, China

**Keywords:** Hepatocellular carcinoma, Alternative splicing, PTBP1, MAPT

## Abstract

**Background:**

Alterations in splicing factors contribute to aberrant alternative splicing (AS), which subsequently promotes tumor progression. The splicing factor polypyrimidine tract binding protein 1 (PTBP1) has been shown to facilitate cancer progression by modulating oncogenic variants. However, its specific role and underlying mechanisms in hepatocellular carcinoma (HCC) remain to be elucidated.

**Methods:**

PTBP1 expression was evaluated in HCC tissues and cell lines. Subsequently, cells were transfected with vectors designed for PTBP1 overexpression or downregulation. The biological function of PTBP1 was assessed *in vitro* and *in vivo* using MTS assays, colony formation assays, transwell assays, xenograft formation, tail vein injection, and orthotopic models. Transcriptome analysis was conducted to elucidate the underlying molecular mechanisms.

**Results:**

Our findings demonstrated that PTBP1 exhibited elevated expression in HCC cell lines and tissues. Furthermore, its expression positively correlated with overall and disease-free survival rates, as well as tumor grade and stage. PTBP1 knockdown reduced HCC cell proliferation, migration, and invasion *in vitro* and suppressed hepatocarcinoma xenograft growth and infiltration *in vivo*. RNA sequencing (RNA-Seq) analysis identified the AS events associated with PTBP1. PTBP1 functionally enhanced cell proliferation, invasion, and migration by modulating the AS of the microtubule-associated protein tau (*MAPT*) gene and promoting oncogene expression. Notably, the dysregulation of MAPT splicing coincided with increased PTBP1 expression in HCC.

**Conclusions:**

PTBP1-guided AS of the *MAPT* gene enhances tumorigenicity in HCC through activation of the MAPK/ERK pathways.

## Introduction

Mounting evidence indicates that alternative splicing (AS) plays a crucial role in protein function [1–5]. Generally, the essential information for splicing is thought to reside in exonic and intronic cis-regulatory elements. These elements function by attracting sequence-specific RNA-binding protein factors, which can either activate or repress the utilization of adjacent splice sites [4,6,7]. Multiple splicing sites can be utilized to generate distinct mRNA isoforms from a single pre-RNA, significantly enhancing protein diversity. The failure to accurately identify splice sites due to splice site mutations or aberrant regulation of splicing factors can result in the production of abnormally mature mRNA variants or harmful isoforms, which substantially contribute to tumor malignancy [8,9]. “Cancerous” splice variants of genes have emerged as potential molecular markers and therapeutic targets for cancer [10,11].

Hepatocellular carcinoma (HCC) ranks as the second leading cause of absolute years of life lost, recognized as a highly aggressive, chronic inflammation-associated cancer [12]. In China, HCC is associated with a high mortality rate [13,14]. HCC is recognized as a highly fatal tumor, with most cases detected at advanced stages, resulting in an incidence-to-mortality ratio approaching 1 [15]. While radical resection is a potential intervention for HCC, it is unsuitable for 50%–70% of patients due to liver dysfunction, advanced tumor stage, or poor performance status [16]. Genomic studies have elucidated the pattern of molecular alterations in HCC. However, the majority of mutations are not actionable, with only approximately 25% of tumors possessing potentially targetable drivers [17]. Consequently, identifying essential markers of HCC and examining their mechanisms are crucial for enhancing therapeutic efficacy and reducing mortality. The complex nature of splicing regulation in the liver suggests that abnormal splicing may play a significant role in hepatocarcinogenesis, although this aspect remains insufficiently explored. Functional investigations have indeed identified multiple splicing factors as potential oncogenic candidates by altering AS products towards tumor-promoting isoforms in hepatocarcinoma cells [18–20].

Polypyrimidine tract binding protein 1 (PTBP1), also known as HNRNP I, is a member of the heterogeneous nuclear ribonucleoprotein (hnRNP) family that regulates mRNA splicing, translation, stability, and localization. Aberrant expression of PTBP1 has been implicated in various diseases. Primarily, PTBP1 modulates AS by controlling precursor mRNA (pre-mRNA) splicing, often resulting in exon skipping. Substantial evidence indicates that PTBP1 is upregulated in numerous malignancies, exerting oncogenic effects through the regulation of AS of genes [21–23]. However, the precise mechanisms of PTBP1’s role in additional cancer types remain inadequately understood, necessitating further comprehensive investigations.

This study aims to characterize the expression, clinical relevance, biological function, and underlying mechanisms of PTBP1 in HCC by focusing on AS control. Higher PTBP1 levels were observed in HCC patients, correlating with adverse outcomes. Through RNA sequencing (RNA-Seq) and subsequent analyses, numerous AS events influenced by PTBP1 were identified, and the position-dependent modulation of AS by PTBP1 in HCC was examined. Furthermore, the study revealed that PTBP1 promotes invasion, migration, and proliferation of HCC cells by altering the AS of microtubule-associated protein tau (MAPT). Collectively, these findings underscore the role of PTBP1 as a splicing regulator in HCC biology, influencing various phenotypic characteristics of HCC.

## Materials and Methods

### Clinical tissue sample collection

Written consent for the utilization of tumor specimens in this research was obtained from the Cancer Hospital of Guangzhou Medical University. The clinical features are described in Table S1. Following surgical resection, the samples were preserved in a 3.7% buffered formaldehyde solution and subsequently encased in paraffin. Sections with a thickness of five micrometers were then prepared and subjected to immunohistochemistry (IHC) for PTBP1 (Abcam, ab133734, Cambridge, UK).

### Cell culture

HCC cell lines HepG2, MHCC97H, MHCC97L, Hep3B, 8024, PLC/PLF/5, Huh7, SK-Hep1, normal human hepatocytes (THLE-2), and the human embryonic kidney cell line HEK293T were obtained from the Cell Bank of the Institute of Oncology, Cancer Hospital, Guangzhou Medical University. The cells were cultured in DMEM (high glucose) (Dulbecco’s Modified Eagle’s Medium, Gibco, 10566-016, New York, NY, USA) supplemented with 10% fetal bovine serum (FBS; Gibco, 10270-106, New York, NY, USA) in a humidified atmosphere containing 5% CO_2_ at 37°C. Mycoplasma contamination was detected by PCR routinely, and cultured cells had no Mycoplasma contamination.

### Animal experiments

The Institutional Animal Care and Use Committee (IACUC) of Guangzhou Medical University approved all animal procedures (Approval Number: 2019-244), ensuring ethical and humane treatment of the animals. All BALB/c nude were purchased from Guangdong Provincial Medical Experimental Animal Center (Foshan, China). Four-week-old female athymic BALB/c nude mice (n = 4 or 5 in each group) were maintained under specific pathogen-free (SPF) conditions. For the mouse xenograft model, cells from the experimental group or the control group were injected subcutaneously into the flank region of the mice at a concentration of 2 million cells. The tumor volumes were calculated using the following equation: volume = (length × width^2^)/2. After euthanasia, the tumor weights were determined. To investigate the effect of PTBP1 or MAPT on tumor metastasis *in vivo*, the researchers injected the experimental group and their corresponding control cells into the liver or tail vein of BALB/c nude mice to induce peritoneal dissemination or pulmonary metastases, respectively. The intensity of the luciferase signal was observed *in vivo* using an *in vivo* Imaging System (FX PRO, Bruker, Billerica, MA, USA). Subsequently, the mice were euthanized, and the presence of metastatic lesions in the abdominal cavity and lungs was evaluated.

### Plasmid assembly and lentiviral packaging

The shRNAs targeting MAPT were acquired from Sangon Biotech Pharma and introduced into HCC cells using Lipofectamine 2000 (Invitrogen). The shRNAs targeting PTBP1 were procured from Jikai Gene Pharma. The sequence of negative control and target shRNAs are listed in Table S2.

### Cell invasion and migration assays

A total of 2 × 10^4^ starved cells in serum-free medium were seeded into a 24-well plate insert with Matrigel (Corning Costar, 353097, New York, NY, USA). The bottom chamber was filled with 20% fetal bovine serum. After 48 h of incubation, invasive cells were fixed with 4% paraformaldehyde, stained with 0.5% crystal violet, counted, and analyzed. The cell migration assay followed the same procedure as the cell invasion assay, with the exception that the upper chamber lacked Matrigel coating.

### MTS assay and colony formation assays

Cells were seeded in 96-well plates (1000 cells per well) and cultured for six days for the MTS assay. Cell proliferation was assessed using the MTS reagent (Promega, G3580, Madison, WI, USA) at 0, 1, 2, 3, 4, 5, and 6 days after incubation. Absorbance was measured at 490 nm using a Multiskan^TM^ FC microplate photometer (Thermo Fisher, Waltham, MA, USA). For the colony formation assay, 1000 cells were plated per well in 6-well plates and cultured for one week. After this incubation, the colonies were fixed, stained, and counted, and images of the colonies were captured. In the soft agar colony formation assay, 2 × 10^3^ cells were mixed with a 0.4% agar solution and seeded in triplicate per well on solidified 0.6% base agar. After 7-day incubation, cell colonies were visualized and imaged using a Leica DMI40008B microscope (Leica, Wetzlar, Germany).

### Western blot analysis

Standard protocols were followed for protein analysis. Total protein was extracted from HCC cells and quantified using the BCA (Thermo Fisher, 23225, USA) assay. Equal protein amounts underwent SDS-PAGE separation and transfer to a polyvinylidene difluoride (PVDF) membrane (Millipore, New Bedford, IPVH00010, USA). The membrane was blocked with 5% skimmed milk, then incubated with primary antibodies overnight at 4°C. Subsequently, it was incubated with secondary antibodies (1:10000) (Thermo Fisher, 616520 and 31450, USA) at room temperature for 2 h. Enhanced chemiluminescence (ECL) (Tanon, 180-501, Shanghai, China) was employed for detection. The antibodies utilized included anti-PTBP1 (1:1000) (Abcam, ab133734, Cambridge, UK), anti-cMYC (1:1000) (Proteintech, 10828-1-AP, Wuhan, China), anti-CyclinD1 (1:1000) (Cell Signaling Technology, 43698, Beverly, MA, USA), anti-ERK1/2 (1:1000) (Cell Signaling Technology, 9102S, Beverly, MA, USA), anti-pERK1/2 (1:1000) (Cell Signaling Technology, 9101S, Beverly, MA, USA), anti-MAPT (1:1000) (Proteintech, 66499-1-Ig, Wuhan, China) and β-actin (Proteintech, 20536-1-AP, Wuhan, China).

### Quantitative real-time PCR analysis (RT-qPCR) and reverse transcription-polymerase chain reaction (RT-PCR)

Following the manufacturer’s guidelines, total RNA was extracted from cultured cells using TRIZOL reagent (Invitrogen, 10296010CN, Carlsbad, CA, USA). According to the manufacturer’s protocol, converted into cDNA using a RevertAid RT kit (Thermo Fisher, k1691, USA). For mRNA detection, gene expression analysis was conducted using the SYBR Green RT-qPCR kit (Takara, RR820Q, Ohtsu, Japan) in accordance with the manufacturer’s instructions. The expression levels were subsequently normalized to GAPDH expression. RT-PCR samples were analyzed using 2% gel electrophoresis. The measurement of each splicing isoform was determined by comparing the integrated optical density of detected bands using Image software (Tanon2500, Shanghai, China). The primers used in this study are listed in Table S2.

### Immunohistochemistry

Mouse tumor slices underwent IHC labeling following established protocols. Antigen retrieval was performed after deparaffinization and rehydration of paraffin-embedded sections. Deparaffinized and redehydrated sections were boiled for 30 min in 1 × citrate buffer and incubated with endogenous peroxidase blockers for 10 min. The primary antibody was applied and incubated overnight at 4°C. Biotin-labeled secondary antibody (Abcam, ab205719 andab97051, Cambridge, UK) to each slide and incubate at room temperature for 10 min. To detect immunoreactivity, the DAB Envision Kit (Maixin, DAB-0031, Fuzhou, China) was used according to the manufacturer’s instructions. The sections were counterstained with haematoxylin (Beyotime, C0107, Shanghai, China). The primary antibodies employed in the experiments were anti-PTBP1 antibody (1:200) (Abcam, ab133734, Cambridge, UK), anti-Caspase3 antibody (1:300) (Cell Signaling Technology, 9664T, Beverly, MA, USA), and anti-Ki67 antibody (1:400) (Cell Signaling Technology, 9449T, Beverly, MA, USA). Caspase-3, an apoptosis marker, indicates whether cells have entered the apoptotic process, while Ki67, a cell proliferation marker, is commonly used to assess cell division activity and analyze tumor growth rates.

### Data collection

The Cancer Genome Atlas Liver Hepatocellular Carcinoma (TCGA-LIHC) data collection is an integral component of a broader initiative aimed at establishing a research community focused on linking cancer phenotypes to genotypes. This is accomplished by providing clinical images corresponding to subjects from The Cancer Genome Atlas (TCGA). Cancer grading refers to the assessment of tumor cell abnormality compared to normal cells under microscopic examination, which determines the degree of histopathological abnormality. The grading system comprises four categories: G1 (well-differentiated), G2 (moderately differentiated), G3 (poorly differentiated), and G4 (undifferentiated). Cancer staging, in contrast, is typically based on multiple factors, including primary tumor size, depth and extent of invasion, involvement of adjacent organs, local and distant lymph node metastasis, and distant metastasis. The predominant method for cancer staging utilizes the TNM classification system.

### Statistical analyses

Statistical analyses were performed using SPSS 23.0 software (SPSS, Chicago, IL, USA) or GraphPad PrismV7 (GraphPad Prism, La Jolla, CA, USA). All results follow a normal distribution. The data were analyzed using various statistical methods, including the Student’s *t*-test, ANOVA, Kaplan-Meier method, log-rank test, Cox regression, and pairwise expression correlation. All quantitative data are presented as mean ± standard deviation. A *p*-value < 0.05 was considered to be statistically significant. *p < 0.05, **p < 0.01, ***p < 0.001, ****p < 0.0001.

## Results

### PTBP1 exhibits elevated expression in HCC tissue and correlates with unfavorable prognosis

**Figure 1:**
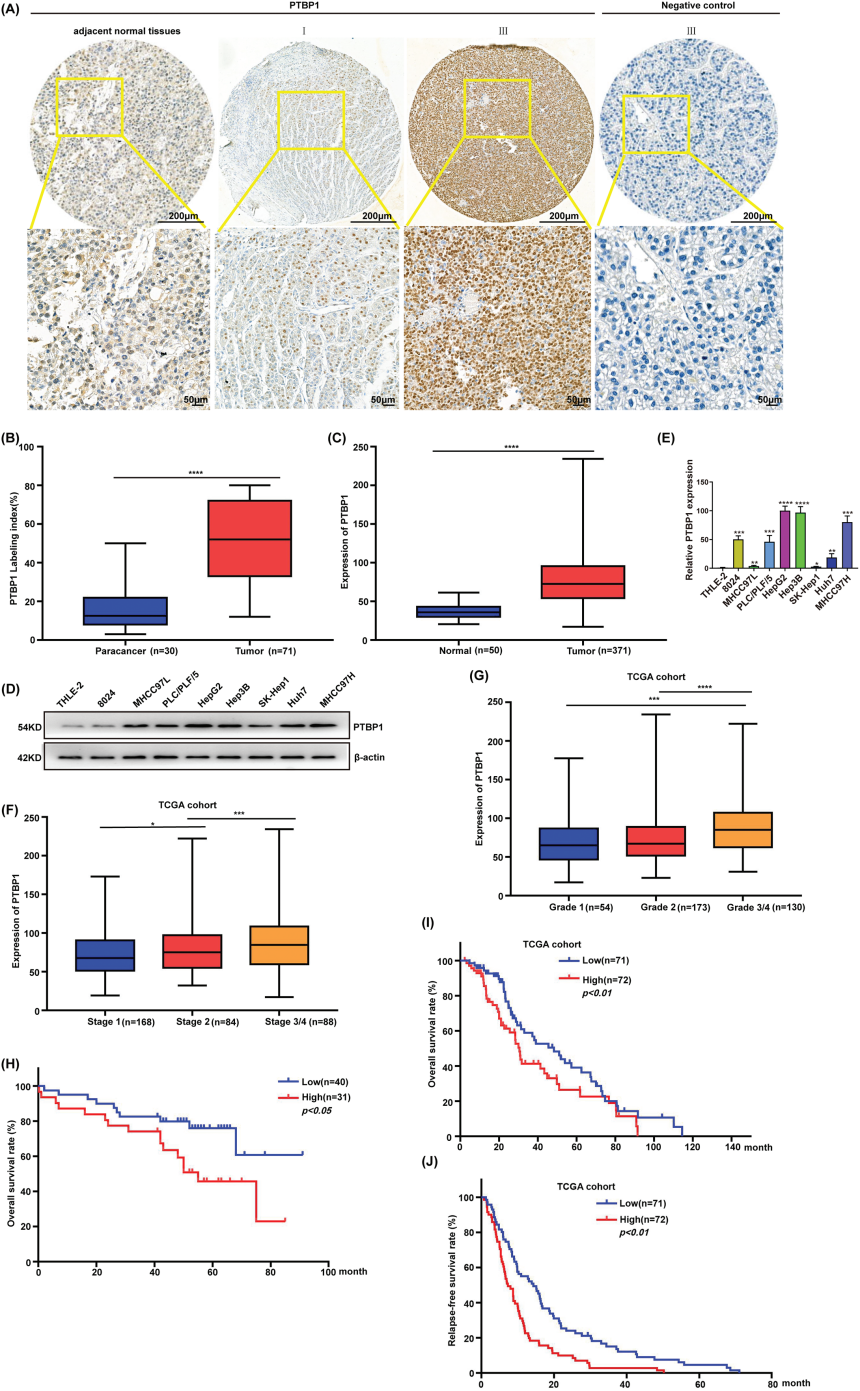
PTBP1 is upregulated in HCC tissue and associated with poor prognosis. (A and B) PTBP1 exhibited higher expression in HCC samples compared to adjacent normal tissues. (A) IHC experiments were conducted on HCC samples and adjacent normal tissues using antibodies against PTBP1. (B) Statistical comparison of PTBP1 levels between HCC samples and adjacent normal tissues. Labeling indices (LIs (%)) serve as a measure of expression levels. Box plots display the data, with lines representing the 25th and 75th percentiles and whiskers indicating minimum and maximum points. ****p < 0.0001 by 1-way ANOVA with Tukey’s posttest. PTBP1 expression in different tumors. (C) PTBP1 expression levels in human HCC tissues (n = 371 independent samples) and noncancerous liver tissues (n = 50 independent samples) from TCGA data. Horizontal lines in box plots represent medians, boxes represent interquartile ranges, and whiskers represent minimum and maximum values. ****p < 0.0001 by Mann–Whitney test. (D) Western blot analysis of PTBP1 expression in HCC cell lines (8024, MHCC97L, PLC/PLF/5, HepG2, Hep3B, SK-Hep1, Huh7 and MHCC97H) and human fetal hepatocyte line (THLE-2). (E) RT-qPCR of PTBP1 expression in HCC cell lines (8024, MHCC97L, HepG2, PLC/PLF/5, Hep3B, SK-Hep1, Huh7 and MHCC97H) and human fetal hepatocyte line (THLE-2). *p < 0.05, **p < 0.01, ***p < 0.001, ****p < 0.0001. (F) PTBP1 expression levels increased in the advanced stages of HCC. PTBP1 levels in the TCGA-LIHC cohort were analyzed. *p < 0.05, ***p < 0.001. (G) PTBP1 showed high expression in HCC. Analysis was performed on PTBP1 levels in the TCGA-LIHC cohort. ***p < 0.001, ****p < 0.0001. (H) Kaplan–Meier analysis of overall survival in PTBP1-high and PTBP1-low groups of our HCC cohort. (I) Kaplan–Meier analysis of overall survival in PTBP1-high and PTBP1-low groups of the TCGA-LIHC cohort. (J) Kaplan–Meier analysis of recurrence-free survival in PTBP1-high and PTBP1-low groups of the TCGA-LIHC cohort.

To validate PTBP1 expression in HCC genesis, we initially employed IHC to examine PTBP1 expression in HCC tissues and corresponding normal tissues. Figs. 1A,B and S3A demonstrated that PTBP1 levels in HCC tissues were elevated compared to the corresponding normal tissues, suggesting an association between PTBP1 and HCC. Furthermore, we investigated PTBP1 expression in these cohorts by analyzing expression profiling data from the TCGA database. We observed significantly higher PTBP1 levels in HCC tissues, corroborating the IHC staining findings (Fig. 1C). PTBP1 upregulation was also evident in HCC cell lines (8024, MHCC97L, PLC/PLF/5, HepG2, Hep3B, SK-Hep1, Huh7 and MHCC97H) compared to the human fetal hepatocyte line (THLE-2) (Fig. 1D,E). Subsequently, we examined PTBP1 presence in various HCC stages and noted a substantial increase in PTBP1 expression in stage III HCC compared to stage I HCC (Fig. 1A), indicating potential PTBP1 elevation in advanced stages. Similarly, we evaluated PTBP1 expression in the TCGA-LIHC cohort’s tumor and normal samples, yielding comparable results (Fig. 1F,G), we categorized HCC cases based on PTBP1 levels. As illustrated in Fig. 1H, Kaplan–Meier analysis revealed that the group with elevated PTBP1 expression exhibited a relatively lower overall survival rate compared to the group with lower PTBP1 expression. The TCGA-LIHC cohort confirmed these observations (Fig. 1I). Correspondingly, the TCGA database indicated poor recurrence-free survival prognosis for patients with high PTBP1 levels (Fig. 1J). A correlation regression analysis revealed an association between stage and high PTBP1 protein levels (Table S1). Collectively, these findings suggest that elevated PTBP1 levels are associated with an unfavorable prognosis for HCC.

### PTBP1 promotes HCC cell proliferation, migration, and invasion

**Figure 2:**
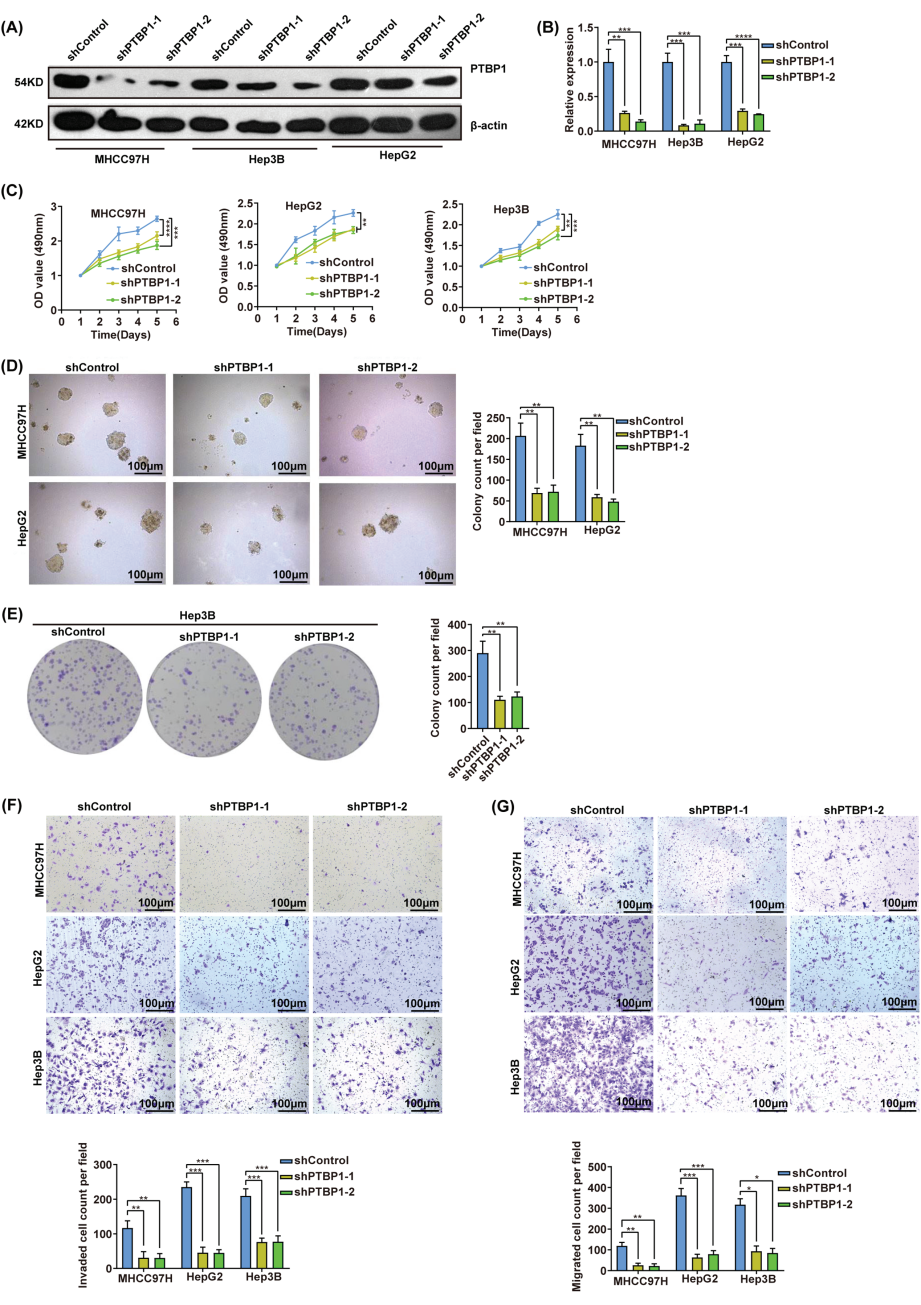
PTBP1 promotes HCC cell proliferation, invasion, and migration. (A and B) Assessment of PTBP1 expression in MHCC97H, Hep3B, and HepG2 cells transfected with shControl or shPTBP1. (C) MTS assay, (D) soft agar colony formation assay, (E) colony formation assay, (F) invasion assay, and (G) migration assay in MHCC97H, Hep3B, and HepG2 cells transfected with shControl or shPTBP1. Scale bars, 100 μm. Error bars represent SD from three independent experiments performed in triplicate. *p < 0.05, **p < 0.01, ***p < 0.001, ****p < 0.0001.

To investigate the role of PTBP1 in HCC, various HCC cell lines, including MHCC97H, HepG2, Hep3B, and SK-Hep1, were transfected with pcDNA3.1(+) vectors containing human PTBP1 inserts or shRNAs. Transfection efficiency was verified through western blot and RT-qRCR (Fig. 2A,B, Fig. S1A–B). To assess whether PTBP1 promotes HCC cell growth, MTS, soft agar cloning assays, and colony formation assays were performed (Fig. 2C–E). As shown in Fig. 2C–E, PTBP1 silencing in MHCC97H, Hep3B, and HepG2 cell lines led to decreased cell proliferation compared to control groups, while PTBP1 overexpression significantly enhanced proliferation (Fig. S1C).

Subsequently, we examined the migratory and invasive characteristics of HCC cells following PTBP1 downregulation or upregulation. The knockdown of PTBP1 significantly impeded the migration and invasion of MHCC97H, Hep3B, and HepG2 cells (Fig. 2F,G), whereas PTBP1 overexpression markedly enhanced the migration and invasion of SK-Hep1 cells (Fig. S1D–E). These *in vitro* cell studies collectively demonstrated that PTBP1 plays a crucial role in cell proliferation, migration, and invasion.

### PTBP1 knockdown inhibits tumor growth

**Figure 3:**
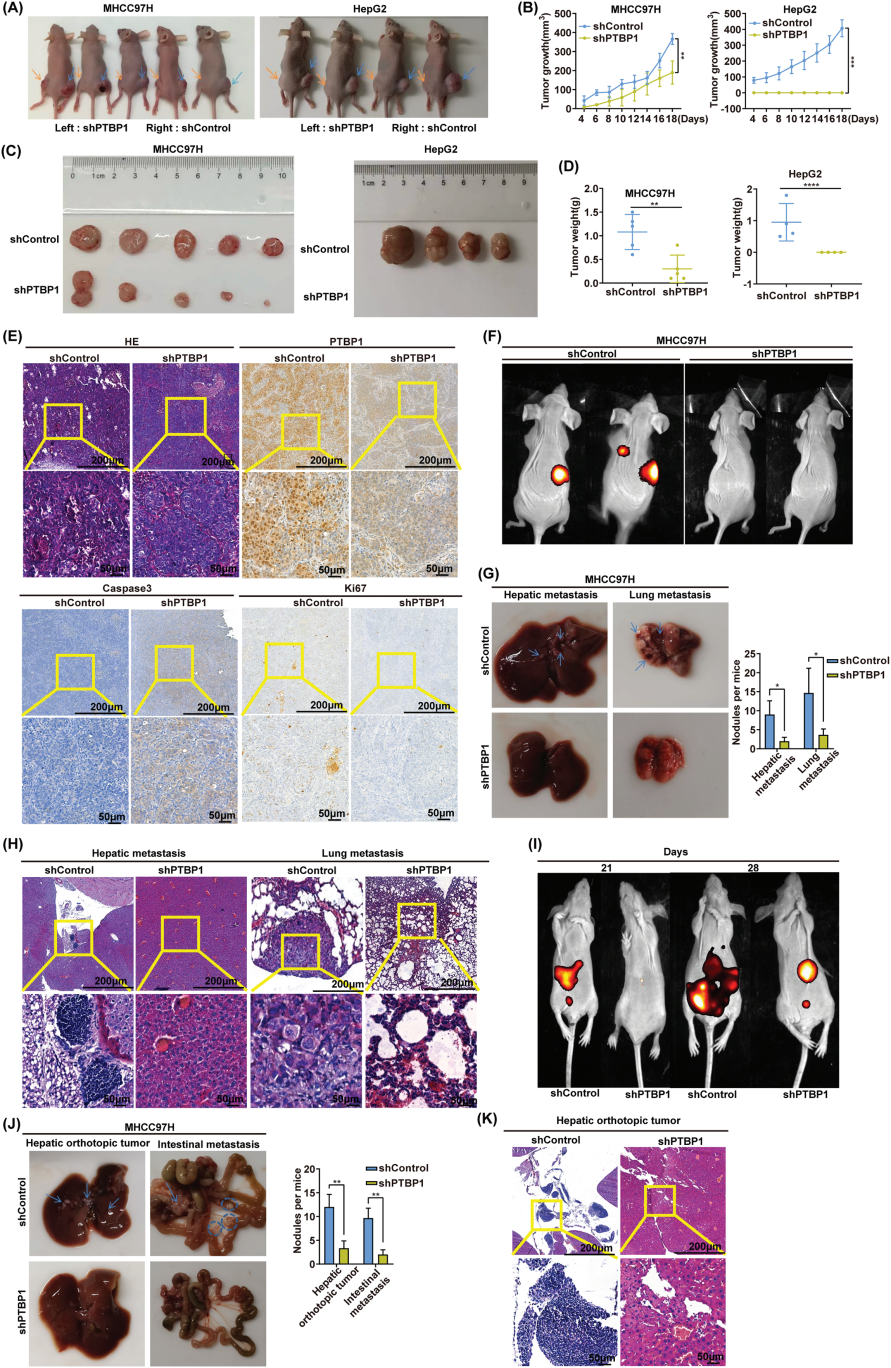
PTBP1 knockdown inhibited tumor growth. (A and C) Representative images of xenograft tumors from shPTBP1-transfected MHCC97H and HepG2 cells in nude mice. (B) Tumor growth curves of shControl or shPTBP1-transfected MHCC97H and HepG2 cells in nude mice over the indicated weeks. **p < 0.01, ***p < 0.001. (D) Tumor weight of each tumor was measured 30 days post-transplantation. Data are presented as mean ± SD. **p < 0.01, ***p < 0.001, ****p < 0.0001. (E) HE staining and IHC were used to analyze the expression of PTBP1, Ki67, and caspase3 in different groups of nude mice. (F–H) MHCC97H cells with stable PTBP1-knockdown or controls (1 × 10^6^ cells) were injected into the tail veins of five nude mice. Lung metastasis luciferase signals, bright-field images, and H&E-stained lung tissue are presented. The number of metastatic foci was quantified. Error bars indicate SD. *p < 0.05. (I–K) Nude mice were intrahepatically injected with 1 × 10^6^ MHCC97H cells stably PTBP1-knockdowned or controls expressing the indicated constructs. After 28 days, the metastasis signal was measured by *in vivo* imaging system, the mice were euthanized, and tumor nodules in the liver and intestine were examined. Bioluminescent, brilliant view, and H&E staining images are displayed. Arrows or circles indicate metastatic nodules. The number of metastatic nodules was quantified. Error bars, SD (*n* = 5 mice/group). **p < 0.01.

To further investigate the role of PTBP1 *in vivo*, we employed a mouse xenograft model to study HCC. PTBP1-depleted cells (shPTBP1-2) were implanted subcutaneously into hairless mice, and tumor growth was monitored over time. As illustrated in Fig. 3A–C, tumor volume was significantly reduced when PTBP1 was knocked down. Furthermore, tumor weight was substantially lower in the PTBP1 knockdown group compared to the control group (Fig. 3D). The number of Ki67-positive cells in the nude mice xenografts decreased markedly due to PTBP1 deficiency, while caspase3-positive cells increased (Fig. 3E, Fig. S3B). Additionally, BALB/c nude mice received stable PTBP1-knockdown MHCC97H cells via intravenous or intrahepatic injection, producing pulmonary metastases, intrahepatic metastasis, or peritoneal dissemination, respectively. In the lung metastasis model, PTBP1 suppression significantly inhibited the metastatic potential of HCC cells to the lungs. This was evidenced by a notable reduction in both the incidence of lung tumors and the number of tumors in the lungs of mice that received PTBP1 knockdown cells (Fig. 3F–H). The intrahepatic metastasis assays revealed fewer metastatic signals in the PTBP1-knockdown group compared to the control group (Fig. 3I–K). Moreover, mice injected with PTBP1-knockdown cells exhibited smaller and fewer tumor nodules in the liver, mesentery, and intestine than the control group. These findings demonstrate that PTBP1 knockdown attenuated the development of HCC tumors *in vivo*.

### Mechanistic exploration of PTBP1-guided AS in HCC

**Figure 4:**
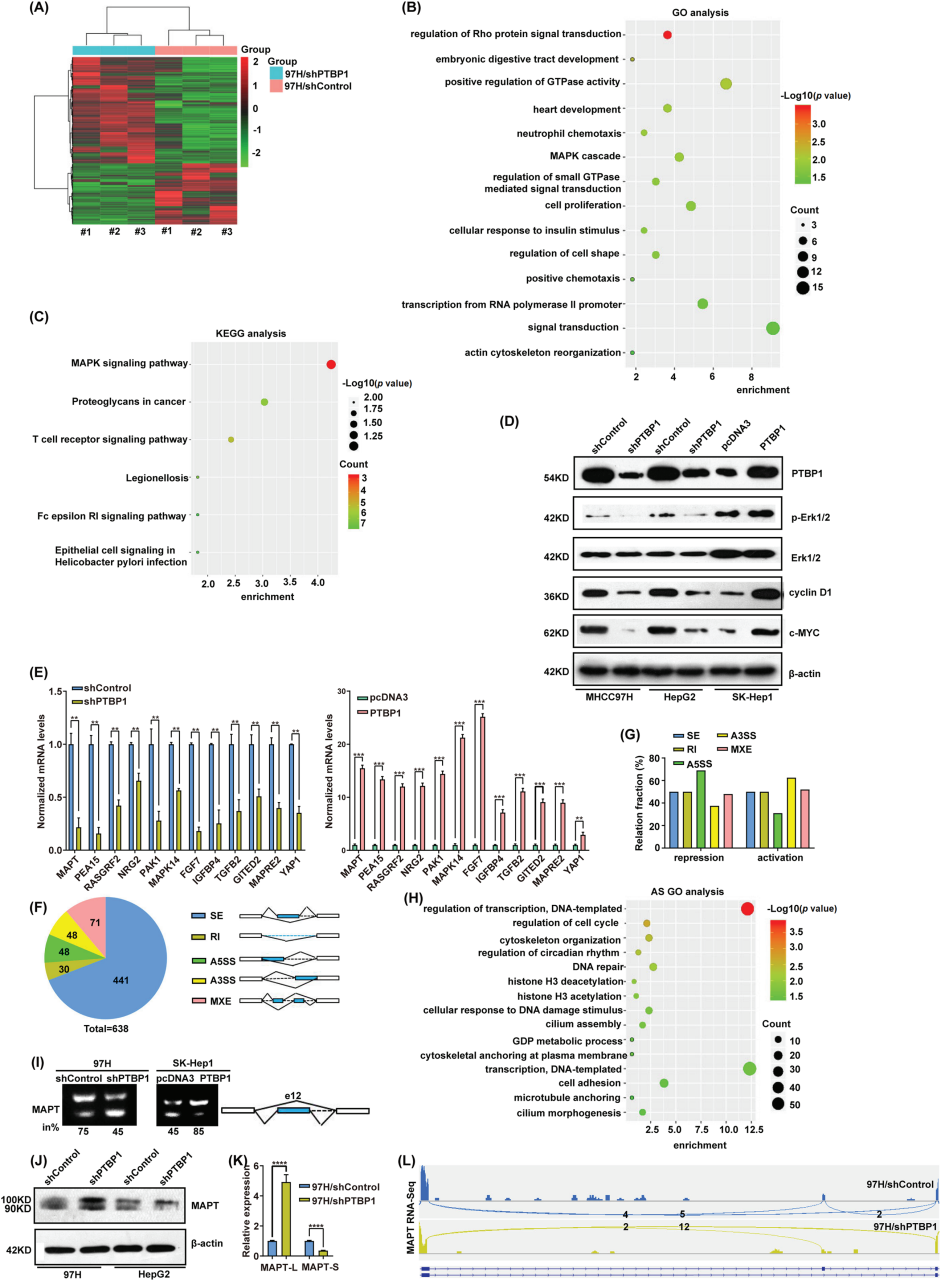
Mechanistic exploration of PTBP1-guided AS in HCC. (A) Heatmap illustrating differentially expressed genes following PTBP1 knockdown. (B) KEGG analysis of enriched pathways in HCC cells with PTBP1 knockdown. (C) Pathway enrichment GO analysis of distinctly expressed genes between shControl and shPTBP1 97H cells. (D) MAPK/ERK signaling pathway marker expression in shControl and shPTBP1 MHCC97H cells. (E) Target gene expression verified by RT-qRCR. (F) PTBP1-affected AS events in MHCC97H cell lines. (G) Relative proportion of AS events affected positively (activation) or negatively (repression) by PTBP1 in each category. (H) GO analysis of enriched pathways in MHCC97H cells with PTBP1-affected AS events. (I) Inclusions of MAPT-L and MAPT-S examined by RT-PCR in MHCC97H, with schematic structure of each PCR product indicated on the right. Alternative exons/introns affected by PTBP1 are highlighted in blue. The percentage of total products that were exon or intron inclusion products (in %) are provided below each gel. (J) and (K) MAPT isoform expression levels examined in PTBP1 stably silenced MHCC97H by Western blot and RT-qRCR. (L) Schematic diagram of RNA-seq reads covering MAPT exon 12 between PTBP1 shRNA stably transduced and control MHCC97H cells. All of the data are representative of at least three independent experiments. Data are presented as the mean ± SD. **p < 0.01, ***p < 0.001, ****p < 0.0001.

To elucidate the molecular mechanism underlying PTBP1’s pro-cancer function, we conducted transcriptomic sequencing on PTBP1-depleted MHCC97H cells and corresponding controls (Fig. 4A). Analysis revealed significant alterations in the expression levels of 518 genes. GO and KEGG analyses indicated that these genes strongly correlated with enhanced GTPase activity, cellular proliferation, signal transduction, and the MAPK/ERK signaling pathway (Fig. 4B,C), further substantiating PTBP1’s role in HCC growth. Western blot results corroborated PTBP1’s activation of downstream MAPK/ERK signaling pathways (Fig. 4D, Fig. S3C). To validate the accuracy of our RNA-Seq findings, we selected 13 distinct genes for verification using stable HCC cell lines with PTBP1 knockdown and overexpression. The results largely aligned with the sequencing data (Fig. 4E).

Subsequently, we analyzed the AS events regulated by PTBP1 and identified 638 AS events that showed significant changes (IncLevelDifference ≥ 0.2, false discovery rate < 0.05). These included 441 cases of skipped exon (SE), 30 cases of retained intron (RI), 48 cases of alternative 3′ splice site (A3SS), 71 cases of mutually exclusive exons (MXE), and 48 cases of alternative 5′ splice site (A5SS) (Fig. 4F–G). The mRNA splicing patterns were observed in genes involved in cell cycle regulation, DNA repair, and response to DNA damage stimuli (Fig. 4H).

To verify the accuracy of our RNA-Seq results, we validated 12 PTBP1-affected AS events (Fig. 4I, Fig. S2). The findings confirmed that PTBP1 exhibited the capacity to either enhance or suppress (Fig. 4G) the splicing of the examined exons/introns.

We specifically examined the *MAPT* gene among the confirmed PTBP1-affected AS events, as its transcripts exhibited a significant shift towards the isoform lacking exons following PTBP1 knockdown (Fig. 4I–K). Initially, we analyzed the expression of exons and identified two primary transcripts: full-length MAPT (containing exon 12, termed MAPT-L) and truncated MAPT (lacking exon 12, termed MAPT-S) (Fig. 4L). The suppression of exon 12 inclusion decreased MAPT protein (MAPT-L) expression and enhanced MAPT-S expression, aligning with the transcriptome sequencing results (Fig. 4I–K).

### MAPT-L isoform enhances the oncogenic capacities of HCC cells

**Figure 5:**
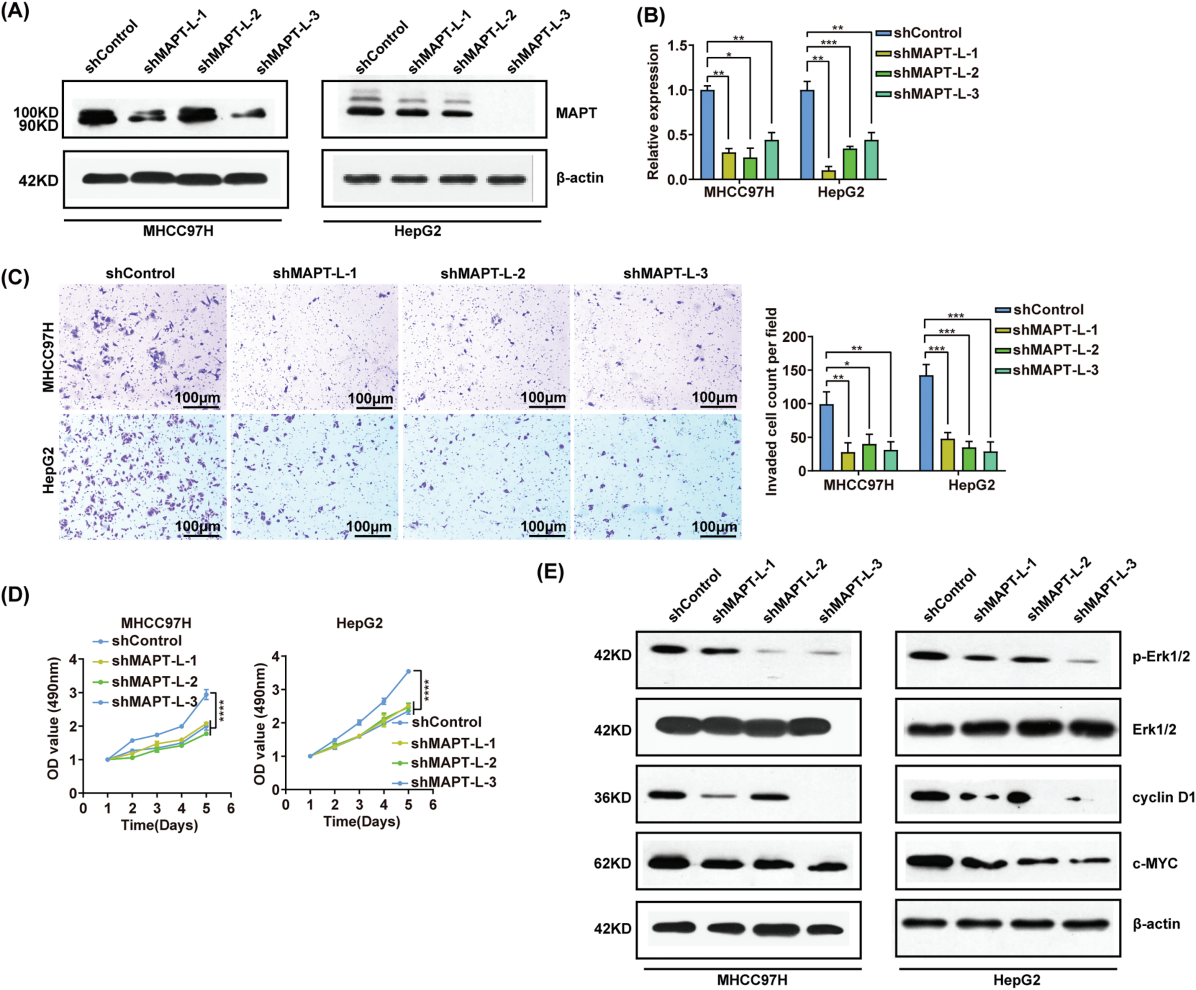
MAPT-L isoform enhances HCC cells oncogenic capacities. (A and B) MAPT-L was efficiently knocked down in MHCC97H and HepG2 cells by MAPT-L shRNA (sh-MAPT-L-1, sh-MAPT-L-2, sh-MAPT-L-3) transfection, as verified by Western blot and RT-qRCR. (C) Transwell invasion assay results. Scale bars, 100 μm. (D) Growth curves of the indicated shRNA-transfected MHCC97H and HepG2 cells. (E) MAPK/ERK signaling pathway marker expression in shControl, shMAPT MHCC97H, and HepG2 cells. Error bars, SD from three independent experiments performed in triplicate. *p < 0.05, **p < 0.01, ***p < 0.001, ****p < 0.0001.

Having established PTBP1’s role in promoting the production of complete MAPT-L, our subsequent investigation focused on elucidating the role and mechanism of MAPT-L in hepatocarcinogenesis. Western blot analysis and RT-qPCR confirmed that three distinct shRNAs targeting exon 12 specifically silenced the MAPT-L isoform (Fig. 5A,B). Stable sub-cell lines expressing control shRNA and MAPT-L shRNA were utilized to determine that MAPT-L knockdown significantly reduced cell invasion (Fig. 5C). MTS assays demonstrated that MAPT-L knockdown effectively inhibited the growth of HCC cells (Fig. 5D). Furthermore, MAPT-L knockdown effectively downregulated cyclinD1 and c-MYC (Fig. 5E, Fig. S3D).

### MAPT isoforms exhibit diverse biological functions

**Figure 6:**
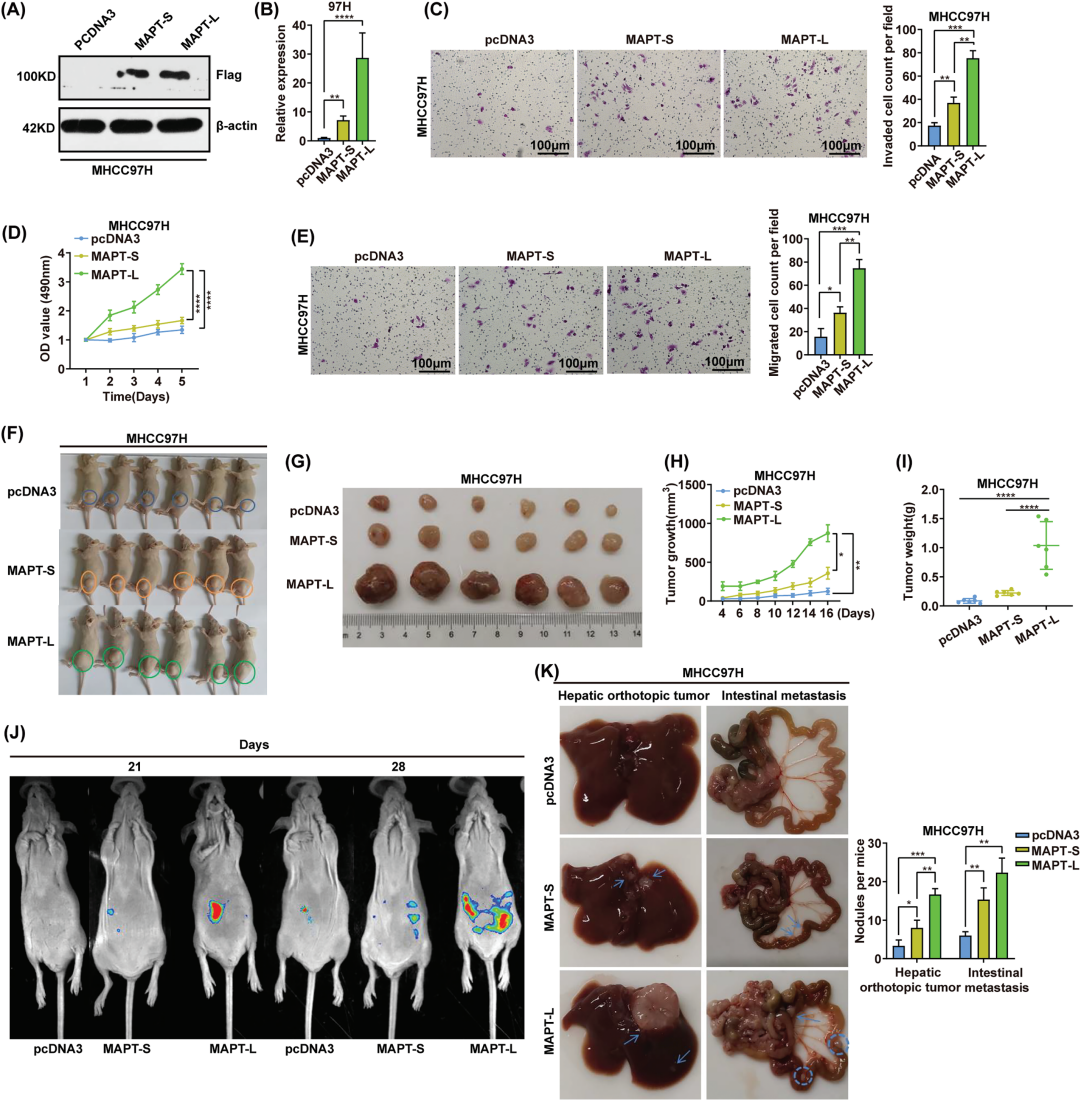
MAPT isoforms exhibit distinct biological functions. (A and B) Western blot and RT-qPCR analyses were conducted to confirm the overexpression of MAPT-L and MAPT-S isoforms in MHCC97H cells. (C and E) Transwell assays were employed to assess the invasive and migratory capabilities of MHCC97H cells stably expressing MAPT-L, MAPT-S, and control cells. (D) MTS assays were performed on MHCC97H cells transfected with MAPT-L or MAPT-S. Scale bars, 10 0 μm. Error bars represent SD from three independent experiments conducted in triplicate. *p < 0.05, **p < 0.01, ***p < 0.001, ****p < 0.0001. (F–I) The effects of MAPT-L or MAPT-S overexpression in MHCC97H cells on subcutaneous tumor growth were evaluated. Images of mice 16 days post-injection are presented. Tumor volumes were measured at 2-day intervals (H). The mice were 21 days post-injection, and the tumors were excised and weighed (G, I). n = 6 mice per group. *p < 0.05, **p < 0.01, ****p < 0.0001. (J and K) 1 × 10^6^ MHCC97H cells stably expressing MAPT-L or MAPT-S were intrahepatically injected into BALB/c nude mice to induce peritoneal dissemination (n = 5 mice/group). Representative bioluminescent and bright-field images are shown. Arrows or circles indicate metastatic nodules. The number of metastatic nodules was quantified. *p < 0.05, **p < 0.01, ***p < 0.001.

Based on the aforementioned findings, we investigated potential differences in the biological functions of the two MAPT isoforms (MAPT-L and -S). To examine the distinct roles of MAPT isoforms (Fig. 6A,B), we established stable overexpression of either MAPT-L or MAPT-S in MHCC97H cells (Fig. 6A,B). Notably, the results demonstrated that the MAPT-L isoform exhibited a more pronounced effect on the proliferation, invasion, and migration abilities of MHCC97H cells, whereas MAPT-S had minimal impact (Fig. 6C,E). In animal trials, the MAPT-L overexpression group displayed increased growth rates of HCC xenografts compared to the control group (Fig. 6F,K). These findings suggest that MAPT-L significantly enhances the growth, motility, and infiltration of HCC cells, while MAPT-S lacks these oncogenic properties.

## Discussion

AS plays a crucial role in gene regulation, yet the precise mechanisms by which the AS machinery is activated or deactivated in malignancies remain largely unknown. This study conducted an extensive investigation into the splicing factor PTBP1 and its subsequent AS landscape in hepatocellular carcinoma. The research identified a critical AS target, MAPT, which demonstrated a strong correlation with the initiation and progression of HCC, as well as patient prognosis. In conclusion, the findings suggest that PTBP1 promotes cancer development by regulating the AS of tumor-associated genes, underscoring the significance of AS as a key regulator of tumor formation.

The human PTBP1 gene is located on chromosome 19p13. This locus frequently demonstrates amplification, which correlates with poor prognoses across various tumor types [24,25]. Moreover, extensive and rigorous studies conducted by other researchers have consistently shown that PTBP1 is significantly overexpressed in diverse malignancies, playing a critical role in oncogenesis by regulating the AS of multiple genes intricately involved in cancer development [26–29]. In this study, we demonstrate that PTBP1 exhibits elevated expression in HCC, and this upregulation is associated with a higher malignancy grade, including histological grade, and a poorer prognosis. Further functional tests confirmed that the reduction of PTBP1 results in a decrease in the *in vitro* proliferation, migration, and invasion of HCC cells, as well as the inhibition of hepatocarcinoma xenograft growth and infiltration *in vivo*. Additionally, the overexpression of PTBP1 enhanced the growth and infiltration of the HCC cell line. These findings suggest that PTBP1 plays a crucial role in promoting hepatocarcinogenesis and may have prognostic value as a biomarker in patients with hepatocarcinoma.

The molecular mechanism by which PTBP1 regulates AS in hepatocarcinoma remains elusive, primarily due to the diverse PTBP1- regulated AS networks implicated in tumorigenesis across different cancer types [30–32]. This study aimed to elucidate this mechanism by employing RNA-Seq to identify significant expression changes and PTBP1-affected AS events in MHCC97H cell lines. The analysis revealed hundreds of such events, with the MAPK/ERK signaling pathway significantly associated with the affected genes. Notably, the SE category emerged as the most prevalent splicing pattern among the AS events observed. PTBP1-regulated genes demonstrated enrichment in pathways controlling cell cycle, DNA repair, and cellular response to DNA damage stimulation. These findings strongly indicate that PTBP1, akin to numerous other splicing factors [33], modulates exon inclusion or exclusion in a position-dependent manner.

Among the multiple AS targets of PTBP1 identified in hepatocarcinoma, this study focused on the MAPT gene due to the significant alteration of its mature mRNA products to the exon-skipped variant following PTBP1 knockdown. Experimental findings revealed that PTBP1 influenced the AS of MAPT pre-mRNA by facilitating the incorporation of exon 12, resulting in the activation of the complete MAPT isoform. The microtubule-associated MAPT gene has been demonstrated to possess diverse functions, including dsDNA stabilization, rDNA heterochromatinization, chromosomal stability maintenance, and potential regulation of nuclear genes [34]. Recently, tau has emerged as a potential indicator for breast and stomach cancers [35–37]. Patients with high Tau showed a trend of poor survival in terms of PFS [38,39]. The nested case-control study showed that the use of raloxifene (targeting MAPT) was associated with 35% reduced breast cancer risk [40]. Previous research has suggested that the absence of MAPT expression may serve as a criterion for identifying gastric and breast cancer patients likely to respond positively to paclitaxel therapy [36,41]. Our study demonstrated that the full-length isoform of MAPT (MAPT-L) exhibited hepatocarcinoma-promoting effects by enhancing HCC cell proliferation, migration, and invasion. Although other mediators may be involved, these data indicate that MAPT-L is a crucial “bridge” molecule mediating PTBP1’s oncogenic effects in HCC. Furthermore, MAPT regulates oncogenic activity through the MAPK/ERK pathway [42]. Based on this information, it can be reasonably inferred that PTBP1 regulates the splice switching of MAPT, thereby contributing to oncogenesis and disease progression.

While our findings elucidate the specific function of PTBP1 in HCC and identify a crucial AS target, MAPT, which demonstrates a strong association with the initiation and progression of HCC and patient prognosis, further investigation is necessary to elucidate MAPT’s function *in vivo*.

## Conclusion

In conclusion, this study elucidates the tumorigenic roles of PTBP1 and the MAPT-L isoform of MAPT in liver cancer cell migration, invasion, and metastasis. The involvement of PTBP1, a splicing factor, in hepatocarcinogenesis and malignant progression is mediated through its facilitation of exon 12 inclusion in MAPT pre-mRNA and subsequent induction of full-length isoform expression. These findings suggest that PTBP1 and MAPT-L have the potential to serve as novel prognostic biomarkers and therapeutic targets for HCC.

## Supplementary Materials

Figure S1 PTBP1 promotes HCC cell growth, invasion, and migration. Assessment of PTBP1 expression in SK-Hep1 cells transfected with pcDNA3.1 and PTBP1(A, B). MTS(C), invasion(D), and migration assays in SK-Hep1 cells transfected with pcDNA3.1 and PTBP1(E). Scale bars, 100 μm. Error bars represent SD from three independent experiments performed in triplicate. *p<0.05, **p<0.01, ***p<0.001.

Figure S2 RT-PCR validation of PTBP1-affected AS events. The structure of each PCR product is schematically represented on the right. Alternative exons/introns influenced by PTBP1 are highlighted in blue.

Figure S3 The quantitative of IHC results and WB. (A) The quantitative of Fig1A. (B) The quantitative of Fig3E. (C) The quantitative of Fig4D. (D) The quantitative of Fig5E. All of the data are representative of at least three independent experiments. In our analysis, ns indicate no significant difference. *p<0.05, **p<0.01, ***p<0.001, ****p<0.0001.

## Data Availability

The authors have uploaded the data to GEO and listed the GEO accession number. Transcriptomics data were downloaded from GEO database under accession number GSE250316.

## Abbreviations

AS: Alternative splicing
PTBP1: Polypyrimidine tract binding protein 1
HCC: Hepatocellular carcinoma
MAPT: microtubule-associated protein tau
MAPK/ERK: Mitogen-Activated Protein Kinase–Extracellular Signal-Regulated Kinase
RNA-Seq: RNA sequencing
Pre-mRNA: precursor mRNA
TCGA-LIHC: The Cancer Genome Atlas Liver Hepatocellular Carcinoma
IHC: Immunohistochemistry
FBS: Fetal bovine serum
IACUC: The Institutional Animal Care and Use Committee
RT-QPCR: Quantitative real-time PCR analysis
RT-PCR: Reverse Transcription-Polymerase Chain Reaction
TCGA: The Cancer Genome Atlas
SE: Skipped exon
RI: Retained intron
A3SS: Alternative 3’ splice site
MXE: Mutually exclusive exons
A5SS: alternative 5’ splice site
MAPT-L: Full-length MAPT, containing exon 12
MAPT-S: truncated MAPT, deleting exon 12

## Supplementary Materials

The supplementary material is available online at https://doi.org/10.32604/or.2025.060958.
