# Supplementary figures and images for: Splicing factor PTBP1 promotes hepatocarcinogenesis via oncogenic splice-switching of MAPT
Source: Oncol Res. 2025 Apr 18;33(5):1121–33. doi: 10.32604/or.2025.060958 (PMC12034000; doi:10.32604/or.2025.060958)

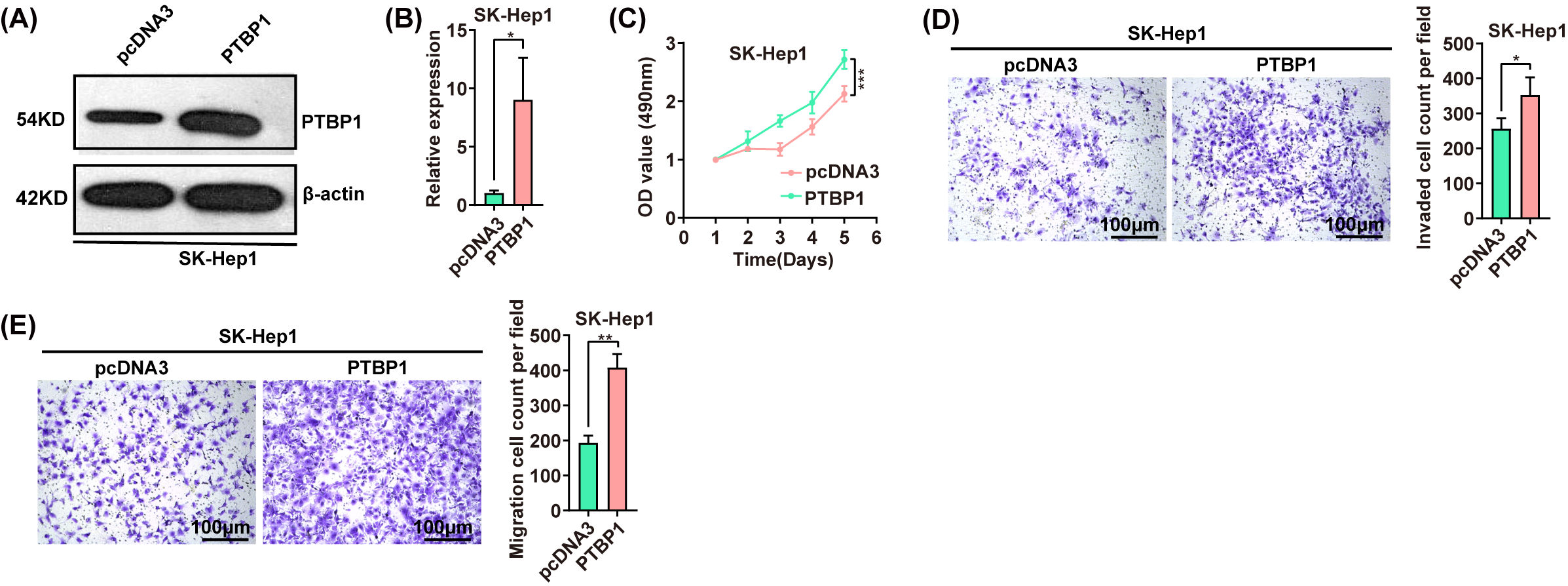

Supplement: Figure S1 [file OncolRes-33-60958-s001.tif]

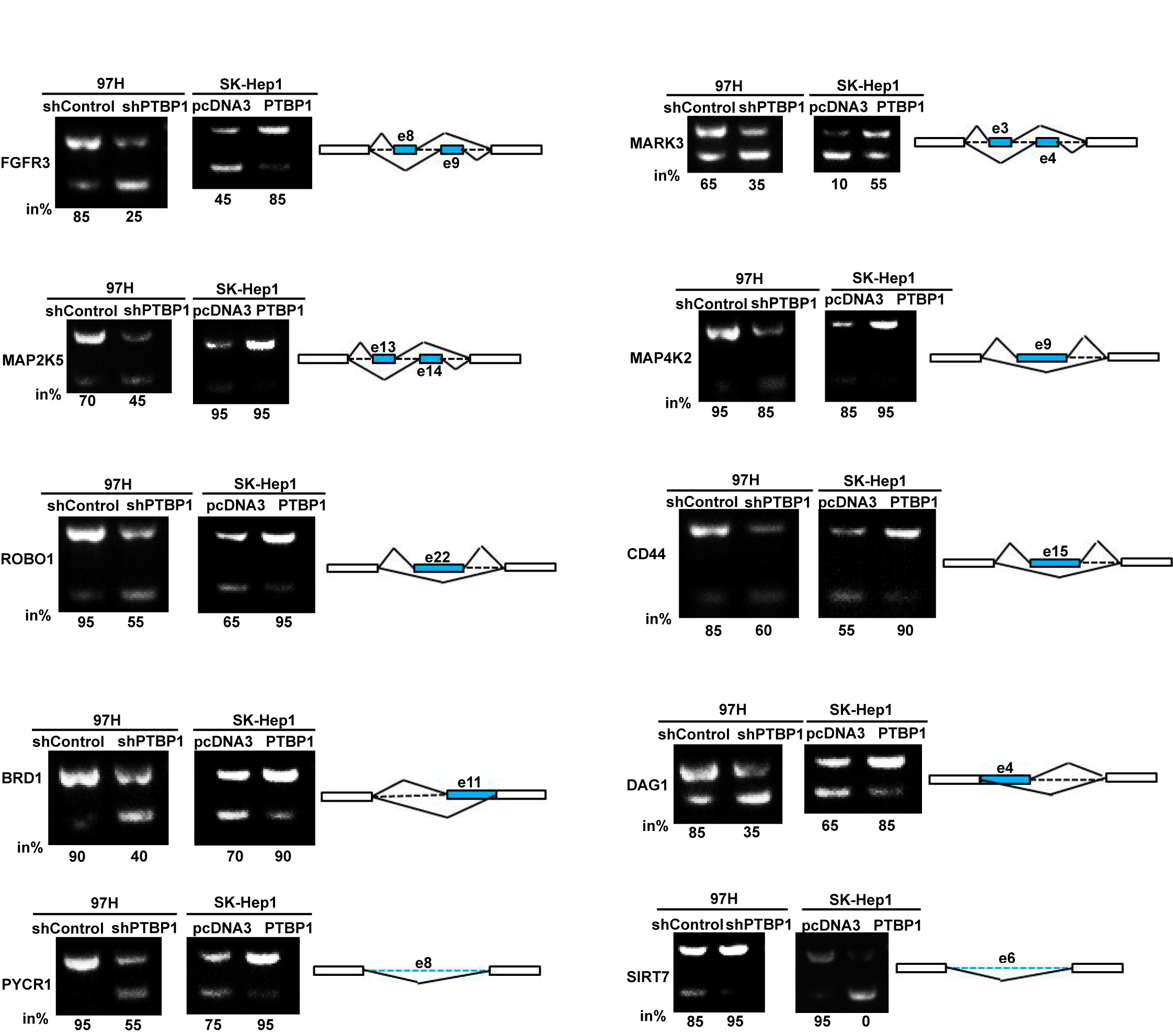

Supplement: Figure S2 [file OncolRes-33-60958-s002.tif]

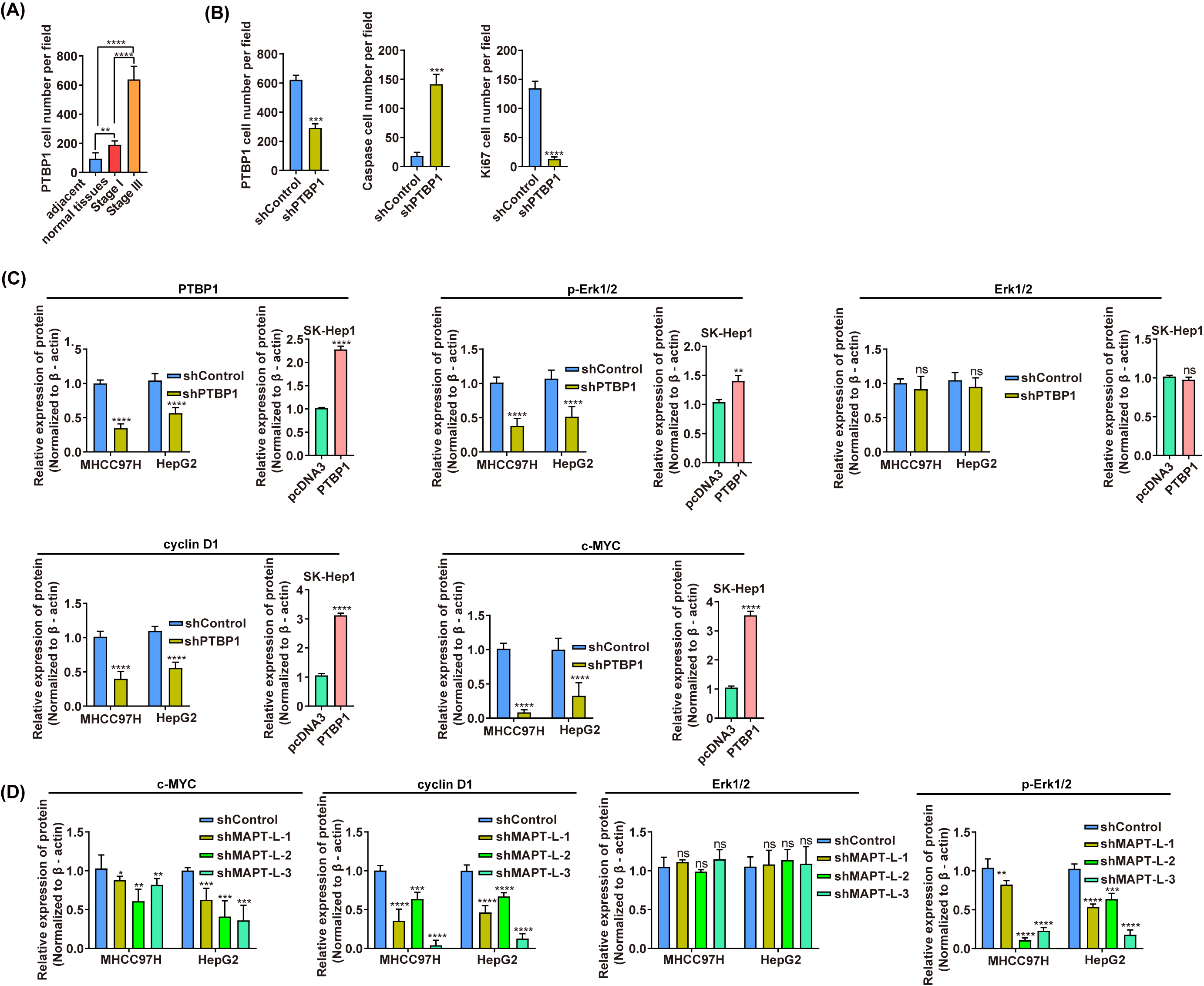

Supplement: Figure S3 [file OncolRes-33-60958-s003.tif]
