# Supplementary material for: Splicing factor PTBP1 promotes hepatocarcinogenesis via oncogenic splice-switching of MAPT
Source: Oncol Res. 2025 Apr 18;33(5):1121–33. doi: 10.32604/or.2025.060958 (PMC12034000; doi:10.32604/or.2025.060958)
Supplement: Supplementary file 4 [file OncolRes-33-60958-s004.docx]

Table S1 Relationship between PTBP1 protein expression and clinicopathological characteristics of the 71 HCC patients

| Parameters | Number | PTBP1 High  （n=31） | | PTBP1 Low  (n=40) | | *p*-value |
| --- | --- | --- | --- | --- | --- | --- |
|  |  | Number | Rate（%） | Number | Rate（%） |  |
| Sex |  |  |  |  |  | 0.662 |
| Male | 61 | 33 | 54.1 | 28 | 45.9 |  |
| Female | 10 | 7 | 70.0 | 3 | 30.0 |  |
| Age |  |  |  |  |  | 0.166 |
| ≥55 | 30 | 16 | 53.3 | 14 | 46.7 |  |
| <55 | 41 | 24 | 58.5 | 17 | 41.5 |  |
| Tumor size(cm) |  |  |  |  |  | 0.120 |
| ≥5 | 48 | 24 | 50.0 | 24 | 50.0 |  |
| <5 | 23 | 16 | 69.6 | 7 | 30.4 |  |
| Grade |  |  |  |  |  | <0.001 |
| Ⅰ | 10 | 9 | 90.0 | 1 | 10.0 |  |
| Ⅱ | 24 | 20 | 83.3 | 4 | 16.7 |  |
| Ⅲ | 37 | 11 | 29.7 | 26 | 70.3 |  |
| AFP (ug/L) |  |  |  |  |  | 0.337 |
| ≥400 | 21 | 10 | 47.6 | 11 | 52.4 |  |
| <400 | 50 | 30 | 60.0 | 20 | 40.0 |  |
| HBV/ HCV  infection |  |  |  |  |  | 0.878 |
| Yes | 59 | 33 | 55.9 | 26 | 44.1 |  |
| No | 12 | 7 | 58.3 | 5 | 41.7 |  |

Note: ****p*< 0.001.
