# Supplementary material for: Splicing factor PTBP1 promotes hepatocarcinogenesis via oncogenic splice-switching of MAPT
Source: Oncol Res. 2025 Apr 18;33(5):1121–33. doi: 10.32604/or.2025.060958 (PMC12034000; doi:10.32604/or.2025.060958)
Supplement: Supplementary file 5 [file OncolRes-33-60958-s005.docx]

Table S2 Primers sequences used for PCR

| **Name** | **Primers (5′ –3′)** |  |
| --- | --- | --- |
| MAPT | Forward  Reverse | CCAAGTGTGGCTCATTAGGCA  CCAATCTTCGACTGGACTCTGT |
| BRD1(exo11) | Forward  Reverse | GTAATCAGTACCAAATGAGAAGGA  AAAGCCGATGGTGAAAC |
| CD44(exo15) | Forward  Reverse | CTCATTGCCCAGCGGACCCCAGACT  CACTGGGGGGGAATGCG |
| DAG1(exo4) | Forward  Reverse | AGGAAGAAACCGACGGAGCGAGGAC  CTGGGAGAGACAGTCGTAT |
| FGFR3(exo8) | Forward  Reverse | GTCACGGAAAGCTGGCGAAGCGAG  AGCGTCACAGCCGCCACAAC |
| MAP4K2(exo9) | Forward  Reverse | GCGTCGCCTCGCGTCGGCTCCGTAG  AGTTTGGGCGTCTGTAA |
| MAP2K5(exo13,14) | Forward  Reverse | GCCTTCATCCTACTCCTCTCGCCAC  AGTGCTAACTCCAAAATCA |
| MAPK3(exo3) | Forward  Reverse | GACTCCGTGAGACTCTGAGGTGCCG  TTCAATTTCAATGACTTCG |
| PYCR1(exo8) | Forward  Reverse | CTAGGTGCAGGTGCCTCGTGACGG  ATGCCTGGACCCTCTGG |
| ROBO1(exo22) | Forward  Reverse | GCACTAATAGTAATACAGATAACGG  TCCCTCCACTGATGACA |
| MAPT (exo12) | Forward  Reverse | GGACCGCCGAGACGCAGGACGCTTG  TCTCAGATTTTACTTCCACC |
| SIRT7(exo6) | Forward  Reverse | CTACCGTGTGAGGCGGAAGCAGAAG  GCGTCCCAGTTCAAAGG |
| GAPDH | Forward  Reverse | AATGGGCAGCCGTTAGGAAA  GCCCAATACGACCAAATCAGAG |
| PTBP1 | Forward  Reverse | GCTGTCATTTCCGTTTGC  GGGTCGGTTCCTGCTATT |
